# α-Asarone blocks 7β-hydroxycholesterol-exposed macrophage injury through blocking elF2α phosphorylation and prompting beclin-1-dependent autophagy

**DOI:** 10.18632/oncotarget.14566

**Published:** 2017-01-09

**Authors:** Sin-Hye Park, Min-Kyung Kang, Yean-Jung Choi, Yun-Ho Kim, Lucia Dwi Antika, Dong Yeon Kim, Eun-Jung Lee, Soon Sung Lim, Young-Hee Kang

**Affiliations:** ^1^ Department of Food Science and Nutrition, Hallym University, Chuncheon, Korea

**Keywords:** a-Asarone, autophagy, ER stress, 7β-hydroxycholesterol, macrophage apoptosis, Pathology Section

## Abstract

Macrophage apoptosis is salient in advanced atherosclerotic lesions and is induced by several stimuli including endoplasmic reticulum (ER) stress. This study examined that a-asarone present in purple perilla abrogated macrophage injury caused by oxysterols *via* ER stress- and autophagy-mediated mechanisms. Nontoxic a-asarone at 1-20 M attenuated 7β-hydroxycholesterol-induced activation of eukaryotic initiation factor 2a in macrophages leading to C/EBP homologous protein (CHOP) expression and apoptosis due to sustained ER stress. The a-asarone treatment increased the formation of autophagolysosomes localizing in perinuclear regions of 7β-hydroxycholesterol-exposed macrophages. Consistently, this compound promoted the induction of the key autophagic proteins of beclin-1, vacuolar protein sorting 34 and p150 responsible for vesicle nucleation, and prompted the conversion of microtubule-associated protein 1A/1B-light chain 3 and the induction of p62, neighbor of BRCA1 and autophagy-related (Atg) 12-Atg5-Atg16L conjugate involved in phagophore expansion and autophagosome formation. Additionally, a-asarone increased ER phosphorylation of bcl-2 facilitating beclin-1 entry to autophagic process. Furthermore, the deletion of Atg5 or beclin-1 gene enhanced apoptotic CHOP induction. Collectively, a-asarone-stimulated autophagy may be potential multi-targeted therapeutic avenues in treating ER stress-associated macrophage apoptosis.

## INTRODUCTION

Autophagy is a catabolic process of self-degradation of cytoplasmic constituents and organelles in the autophagolysosomes [1, 2]. Many autophagy-related genes (Atg) have been identified as components required for optimal autophagic functions [3, 4]. Stepwise autophagic process starts with the engulfment of organelles or portions in the cytoplasm by formed isolation membrane phagophores, which subsequently sequesters cytoplasmic materials into a double-membrane autophagosomes [5]. The class III phosphatidyinositol-3 kinase (PI3K) complex with vacuolar protein sorting 34 (Vps34) mediates the nucleation of the phagophore membrane [3, 4]. This nucleation is blocked by bcl-2 through binding to beclin-1, a component composing PI3K complex. Phagophore membrane elongation and autophagosome formation requires both Atg12-Atg5-Atg16L and microtubule-associated protein 1 light chain 3 (LC3)-phosphatidylethanolamine (PE) conjugates *via* two ubiquitin-like conjugation pathways, along with membrane-bound Atg9. Eventually, these autophagosomes fuse with lysosomes in aid of the LC3-phosphatidylethanolamine conjugate, ultimately leading to formation of the autolysosome. Upon vesicle completion, most of the Atg proteins are dissociated from the autophagosome, allowing autophagosome-lysosome fusion and autophagic cargo degradation [3, 4, 5].

Malfunction of autophagy has been implicated in a variety of diseases and pathologies, including cancer, neurodegeneration, aging and infectious diseases [2, 5, 6]. Autophagy is induced in response to stressors such as nutrient starvation, growth factor deprivation, organelle damage, and endoplasmic reticulum (ER) stress for the maintenance of cellular homeostasis and cell survival [2, 7]. Accordingly, pathogenic alterations in the autophagic machinery have emerged as key targets in the development of novel therapeutic strategies. Pharmacologic agents and molecules have been shown to be capable of influencing distinct steps of autophagic process and targeting the regulatory mechanisms of autophagy [8]. Accordingly, pharmacological approaches to influence autophagic pathways are currently receiving considerable attention for therapy in diseases [9].

Disruption in the normal function of the ER results in cell stress response known as the unfolded protein response (UPR) in response to the accumulation of unfolded proteins [10, 11]. The UPR induces the synthesis of chaperones and protein components for the folding of ER client proteins, initially aiming at compensating for cell injury [12]. When the ER stress is extensive or sustained and the function of ER cannot be restored, the UPR can eventually prompt cell death [13]. There are accumulating data indicating that ER stress is a potent trigger of autophagy [14, 15, 16]. The signaling pathways governing autophagy and the cellular consequences in response to ER stress have been emphasized for the treatment of the numerous diseases related to ER stress [11, 15-17]. However, the physiological and pathological relevance of ER stress-induced autophagy remain puzzling. Emerging data show that ER stress-induced autophagy counterbalances the ER expansion, removes protein aggregates or mutant proteins from the ER [14, 18]. For instance, enhanced autophagy in rapamycin-treated cells reduces the accumulation of the mutant proteins in the ER [19-21]. Accordingly, pharmacological agents enhancing autophagy may display therapeutic possibilities for their clinical exploitation, while autophagy inhibition is being suggested as a strategy for treating some cancers [9].

**Figure 1:**
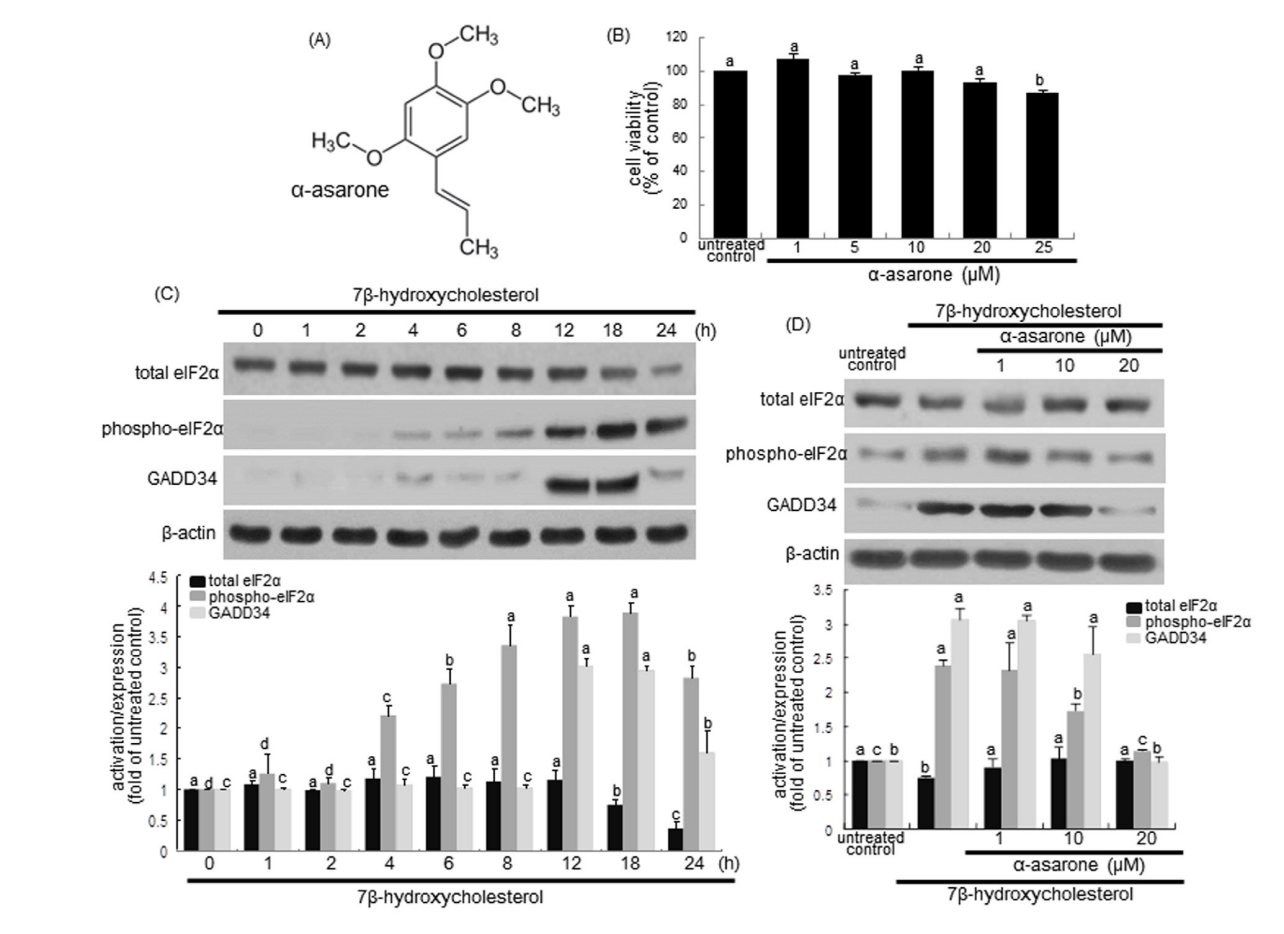
**α-Asarone chemical structure A., macrophage cytotoxicity by α-asarone B., temporal responses of eIF2α expression and activation, and GADD34 induction to 7β-hydroxycholesterol C. and their inhibition by α-asarone D**. J774A1 macrophages were incubated with 28 μM 7β-hydroxycholesterol up to 24 h. Macrophage viability (mean ± SEM, *n* = 5) was measured by using MTT assay and expressed as percent cell survival relative to glucose controls **B**. Cells were lysed, electrophoresed on 12% SDS-PAGE and subject to western blot analysis with a primary antibody against eIF2α, phospho-eIF2α or GADD34 **C**. Macrophages were incubated with 28 μM 7β-hydroxycholesterol in the absence and presence of 1-20 μM α-asarone for 18 h **D**. β-Actin protein was used as an internal control. The bar graphs (mean ± SEM, *n* = 3) in the bottom panels represent quantitative results obtained from a densitometer. Values in bar graphs not sharing a letter indicate significant different at *P* < 0.05.

Natural polyphenolic compounds found in diet, such as genistein, quercetin, curcumin, and resveratrol, can trigger autophagy-associated cell death in cancer through influencing autophagic machinery at various stages [22]. Epigallocatechin-3-gallate (EGCG), a green tea polyphenol, stimulates hepatic autophagy and lipid clearance, possibly contributing to beneficial effects in reducing hepatosteatosis [23]. In addition, this compound reduces ectopic lipid accumulation in vascular endothelial cells through stimulating a mechanism involving autophagy [24]. The current study investigated that α-asarone stimulated autophagy mediated by ER stress in 7β-hydroxycholesterol-exposed macrophages. α-Asarone (Figure 1A) is a component of certain essential oils found in herbal plants and has been isolated in purple perilla extracts [25]. This compound has been shown to be neuroprotective in mice and to reduce LDL cholesterol levels in rats [26, 27]. Our previous study showed that α-asarone inhibited 7β-hydroxycholesterol-induced macrophage apoptosis through blocking ER stress-specific signaling involving caspase activation and C/EBP homologous protein (CHOP) induction [28]. This study examined the involvement of eukaryotic initiation factor 2α (elF2α)-CHOP-growth arrest and DNA damage-inducible protein 34 (GADD34) pathway in oxysterol-triggered beclin-1 activation leading to autophagolysosome formation.

## RESULTS

### Inhibition of eIF2α phosphorylation and GADD34 expression by α-asarone

ER stress promotes eIF2α activation leading to inhibition of protein synthesis, and induces GADD34 protein in cells experiencing environmental and metabolic stress [29]. The current study examined whether the oxysterol 7β-hydroxycholesterol induced the eIF2α phosphorylation and GADD34 expression in macrophages, which was inhibited by non-toxic α-asarone. As shown in Figure 1C, 28 μM 7β-hydroxycholesterol activated eIF2α and induced GADD34 expression in a temporal manner, evidenced by western blot analysis. The eIF2α phosphorylation was elevated from 4 h after the exposure of macrophages to 7β-hydroxycholesterol and reached considerably high at 18-24 h post-exposure (Figure 1C). The protein GADD34 induction was shortly increased at 12-18 h after the challenge of 7β-hydroxycholesterol to macrophages. On the contrary, the eIF2α phosphorylation and GADD34 induction were inhibited by treating macrophages with α-asarone (Figure 1D). Therefore, these results indicate that 7β-hydroxycholesterol induced ER stress leading to the elevation of eIF2α phosphorylation and subsequent GADD34 expression, which was attenuated by submicromolar α-asarone.

### Autophagolysosome formation by α-asarone

**Figure 2:**
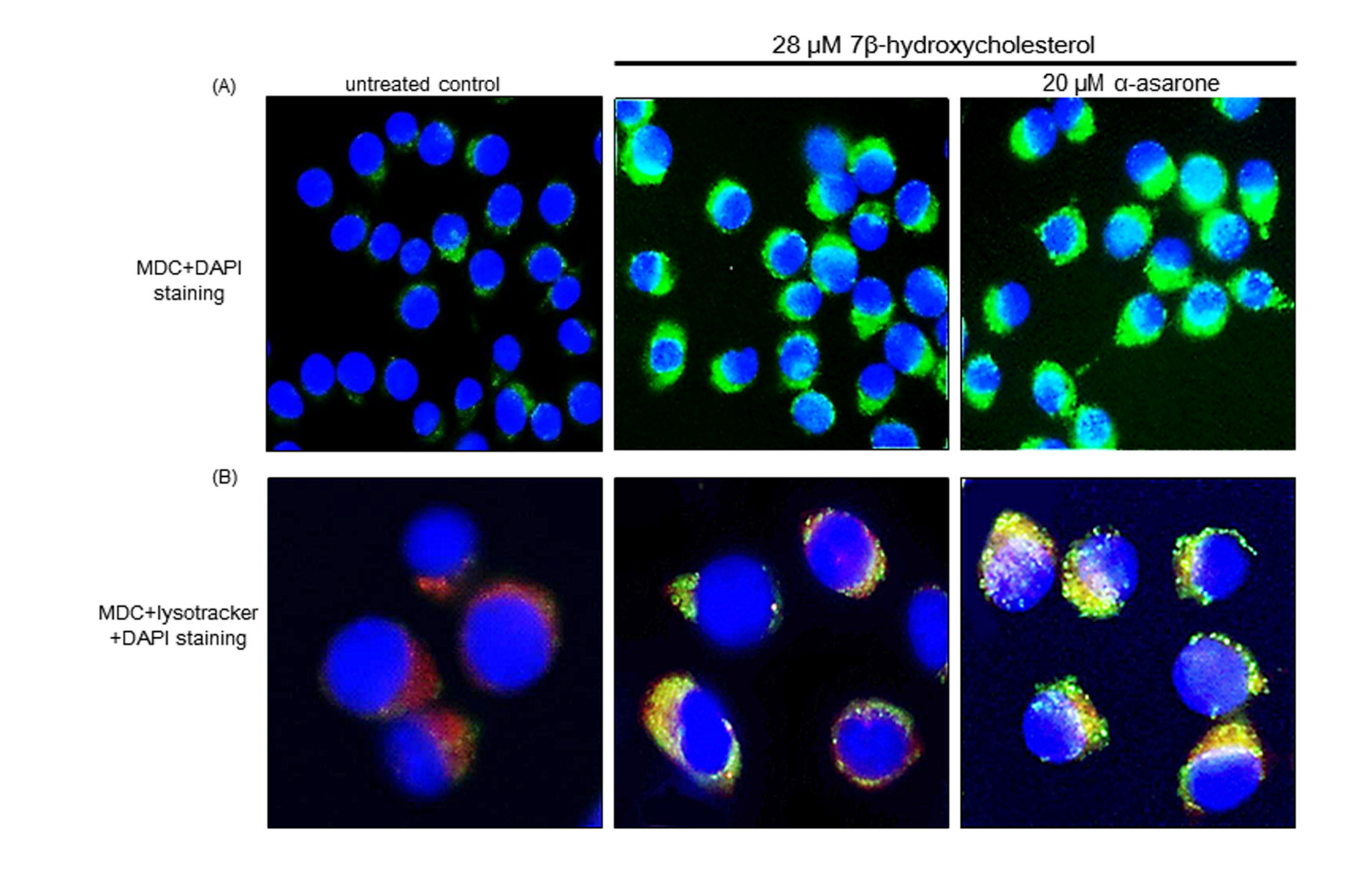
Boosting effects of α-asarone on formation of autophagolysosomes After the macrophages were incubated with 20 μM α-asarone and 28 μM 7β-hydroxycholesterol for 18 h, cells were fixed with 4% paraformaldehyde. J774A1 cells were incubated with 50 μM MDC **A**. and lysotracker **B**. for 1 h at 37°C for the detection of autophagolysosomes. For the nuclear counter-staining, cells were incubated with DAPI for 10 min. Stained cells mounted on slide glass were visualized by fluorescence confocal microscopy.

Emerging evidence suggests that ER stress is a potent inducer of autophagy contributing to cell survival and the disturbance of autophagy rendered cells vulnerable to ER stress [14, 15]. The current study investigated that 7β-hydroxycholesterol stimulated autophagosome maturation, as evidenced by staining with MDC for autophagosomal vacuoles. When 7β-hydroxycholesterol-exposed macrophages were visualized with a fluorescence microscope, autophagic vacuoles such as autophagosomes stained by MDC appeared as distinct green dot-like structures distributed within the cytoplasm or localizing in the perinuclear regions (Figure 2A). There was an increase in the number of MDC-labeled vesicles at 18 h after 20 μM α-asarone treatment, indicating an induction of autophagosome maturation by α-asarone. This study further examined whether α-asarone activated autophagosomal lysosomal fusion by double staining with MDC and the lysosome red stain lysotracker (Figure 2B). There was lack of green MDC staining in untreated controls, whereas autophgophore vacuoles and lysosomes were colocalized in 7β-hydroxycholesterol-exposed macrophages. When α-asarone was supplied to these cells, much stronger yellow staining with a punctate pattern was observed (Figure 2B). These results indicate that α-asarone promoted autophagolysosome formation by 7β-hydroxycholesterol.

### Promotion of autophagy initiation by α-asarone

**Figure 3:**
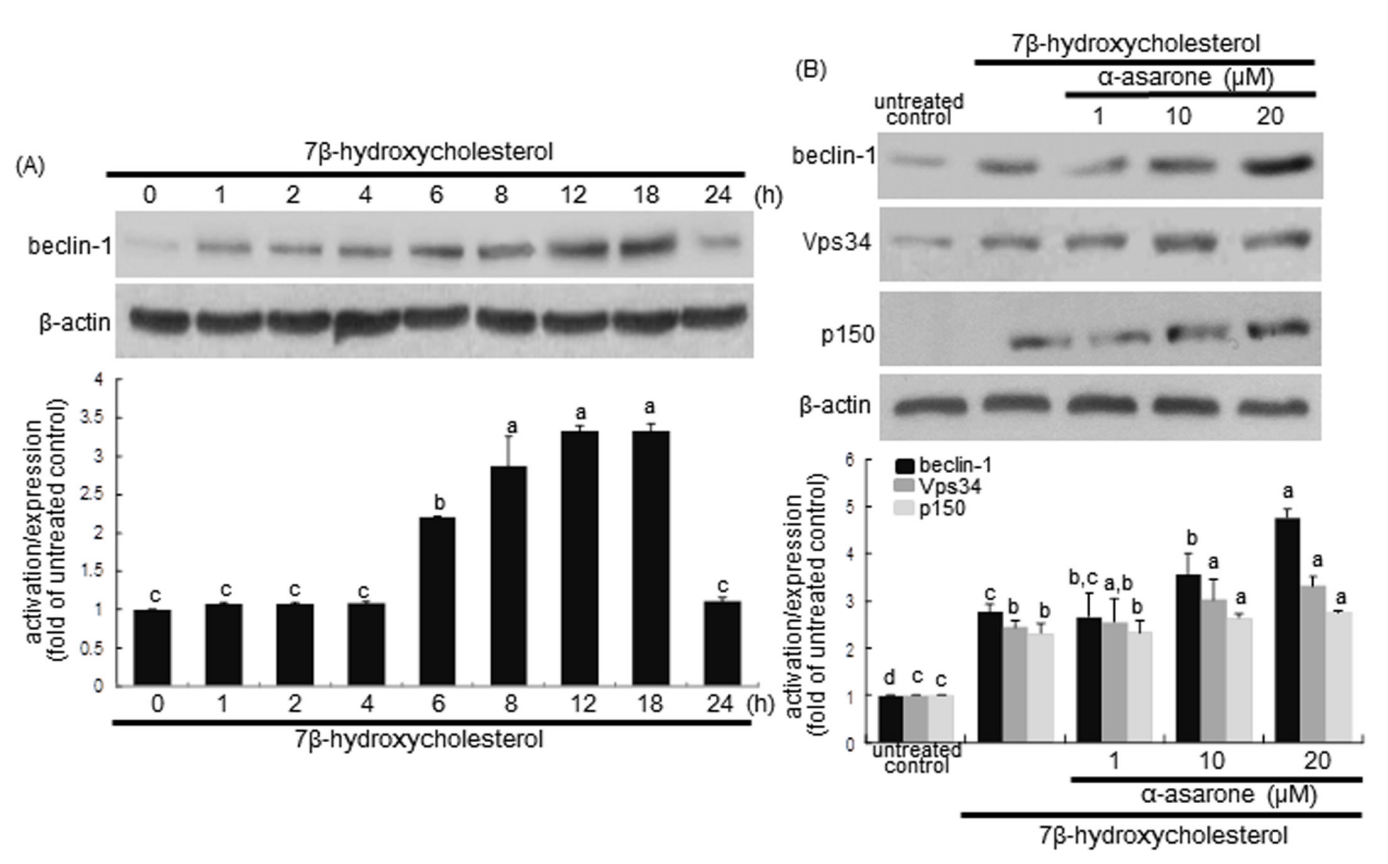
**Western blot analysis showing temporal responses of beclin-1 induction to 7β-hydroxycholesterol A. and potentiation of becin-1, Vps34 and p150 by α-asarone B**. J774A1 macrophages were incubated with 28 μM 7β-hydroxycholesterol up to 24 h. Cells were lysed and electrophoresed on 8% SDS-PAGE, followed by western blot analysis with a primary antibody against beclin-1, Vps34 or p150. Cells were exposed to 28 μM 7β-hydroxycholesterol in the absence and presence of 1-20 μM α-asarone for 18 h **B**. β-Actin protein was used as an internal control. The bar graphs (mean ± SEM, *n* = 3) in the bottom panels represent quantitative results obtained from a densitometer. Values in bar graphs not sharing a letter indicate significant different at *P* < 0.05.

Beclin-1 is a mammalian ortholog of yeast Atg6 and a core component of the autophagy machinery as a part of type III phosphatidylinositol 3 (PI3) kinase complex that is required for initiating the autophagic vacuole formation, so called as vesicle nucleation [30]. Western blot analysis revealed that the beclin-1 levels were gradually increased from 1 h after 7β-hydroxycholesterol treatment and sustained high up to 24 h (Figure 3A). When ≥10 μM α-asarone was treated to 7β-hydroxycholesterol-exposed macrophages, the beclin-1 induction was further enhanced, indicating that α-asarone accelerated autophagy process induced by oxysterol (Figure 3B). In addition, the induction of Vsp34 and p150 proteins increased in a similar manner (Figure 3B). Accordingly, α-asarone improved autophagic nucleation in oxysterol-stimulated macrophages.

**Figure 4:**
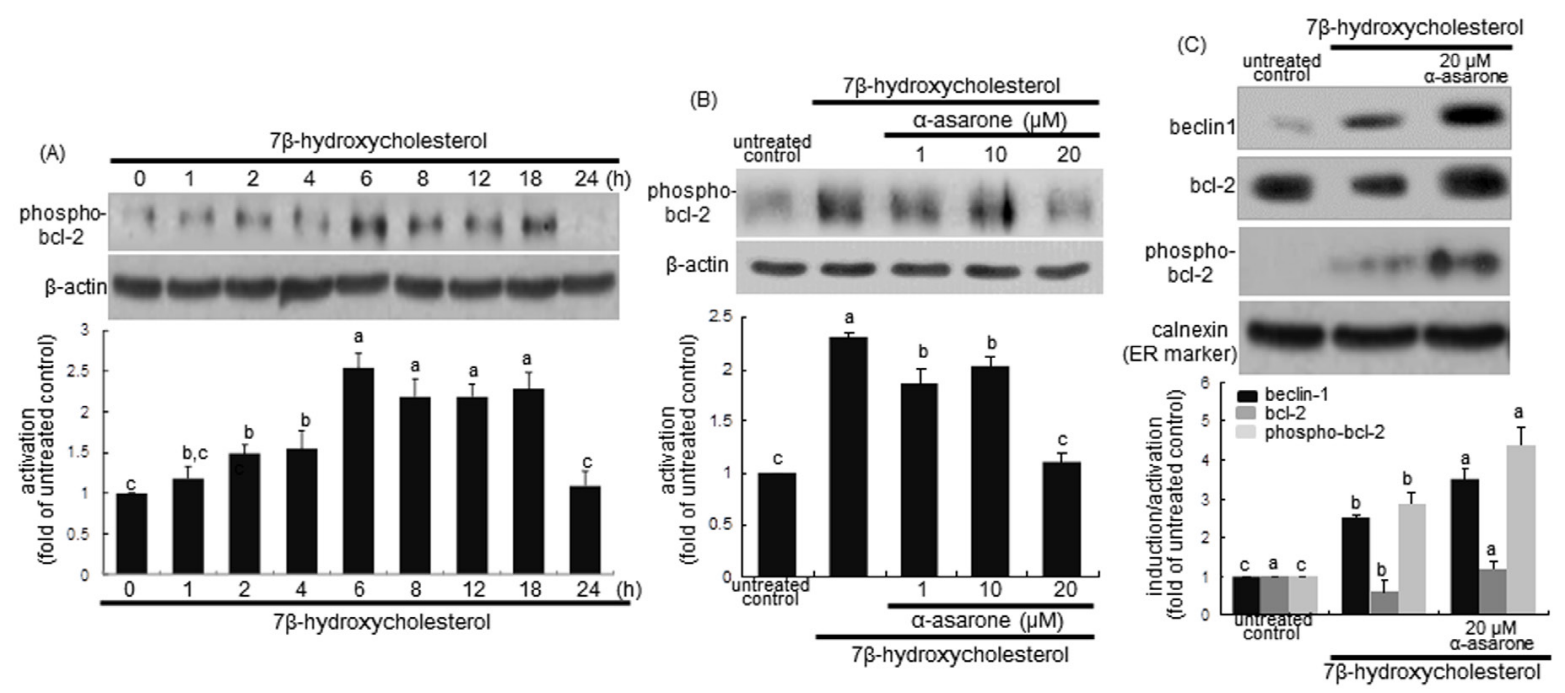
**Western blot analysis showing temporal responses of bcl-2 phosphorylation to 7β-hydroxycholesterol A., its inhibition by α-asarone B., and beclin-1 induction and bcl-2 phosphorylation in ER C**. J774A1 macrophages were incubated with 28 μM 7β-hydroxycholesterol up to 24 h **A**. Cells were exposed to 28 μM 7β-hydroxycholesterol in the absence and presence of 1-20 μM α-asarone for 18 h **B**. The ER-enriched extracts were obtained by using a commercial kit **C**. Cell lysates and ER extracts were electrophoresed on 8% SDS-PAGE, followed by western blot analysis with a primary antibody against phospho-bcl-2, beclin-1 or bcl-2. β-Actin and calnexin proteins were used as internal controls. The bar graphs (mean ± SEM, *n* = 3) in the bottom panels represent quantitative results obtained from a densitometer. Values in bar graphs not sharing a letter indicate significant different at *P* < 0.05.

There is growing evidence that at the ER beclin-1 interacts with anti-apoptotic bcl-2 family proteins, hence attenuating autophagy activity [31]. The disruption of beclin-1-Vps34 complexes can be achieved by bcl-2 phosphorylation by c-Jun N-terminal kinase 1 or beclin-1 phosphorylaion by death-associated protein kinase [31]. The bcl-2 activation was considerably enhanced from 6 h after the treatment with 7β-hydroxycholesterol (Figure 4A). Unexpectedly, ≥10 μM α-asarone inhibited cellular phosphorylation of bcl-2 in 7β-hydroxycholesterol-exposed macrophages (Figure 4B). This study further examined whether α-asarone activated autophagy through blocking the binding of beclin-1 and bcl-2 in the ER of 7β-hydroxycholesterol-treated macrophages. 7β-Hydroxycholesterol declined the bcl-2 level in ER, while 20 μM α-asarone restored the induction, possibly increasing anti-apoptotic activity (Figure 4C). The bcl-2 phosphorylation increased in the ER-enriched extracts in response to α-asarone in a similar manner to beclin-1 induction (Figure 4C). These results indicate that this compound boosted the autophagic activity through relieving bcl-2-mediated repression of beclin-1-Vps34 complexes in 7β-hydroxycholesterol-exposed macrophages.

### Elevation of LC3 lipidation and p62/sequestosome 1 (SQSTM1) induction by α-asarone

**Figure 5:**
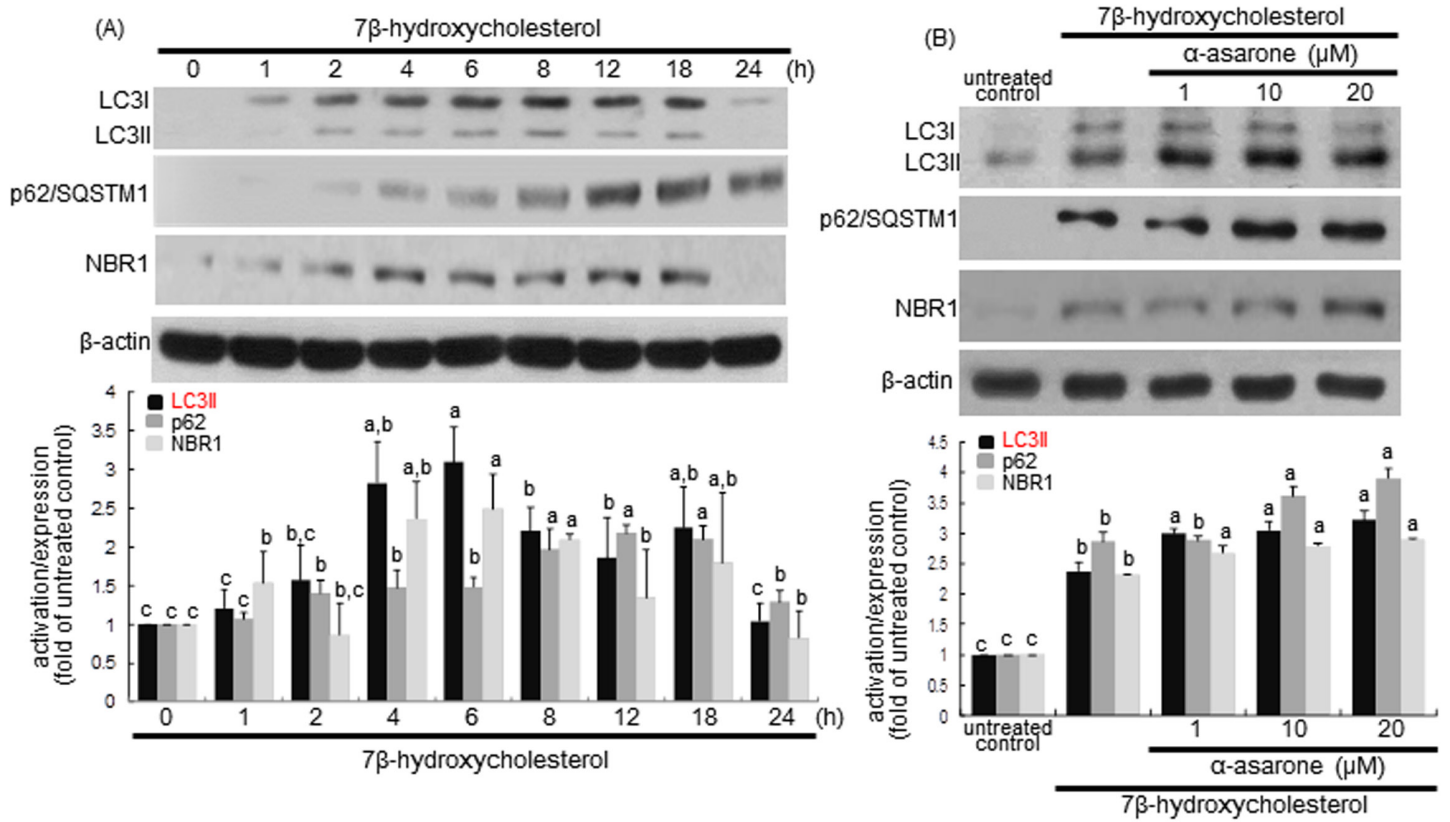
**Time course responses of induction of LC3, p62/SQSTM1 and NBR1 to 7β-hydroxycholesterol A. and promoting effects of α-asarone on their induction B**. J774A1 cells were incubated with 28 μM 7β-hydroxycholesterol up to 24 h **A**. Cells were lysed, electrophoresed on 15% SDS-PAGE, and subject to western blot analysis with a primary antibody against bcl-2, LC3, p62/SQSTM1, or NBR1. Cells were exposed to 28 μM 7β-hydroxycholesterol in the absence and presence of 1-20 μM α-asarone for 18 h **B**. β-Actin protein was used as an internal control. The bar graphs (mean ± SEM, *n* = 3) in the bottom panels represent quantitative results obtained from a densitometer. Values in bar graphs not sharing a letter indicate significant different at *P* < 0.05.

LC3 is localized in autophagosome membranes and is considered as a molecular marker of phagophores and autophagosomes. The cytoplasmic form of LC3I is converted into autophagosome membrane-bound lipidated form of LC3II that is correlated with the extent of autophagosome formation [32]. Using western blot analysis with a LC3 antibody, this study examined the LC3I and LC3II modification in macrophages after treatment with 7β-hydroxycholesterol for 24 h. The LC3II conversion was significantly elevated from 2 h after treatment with 7β-hydroxycholesterol, with a sustained effect occurred up to 18 h (Figure 5A). When 1-20 μM α-asarone was treated to macrophages exposed to 7β-hydroxycholesterol for 18 h, a further increase in the levels of LC3II protein was apparently observed (Figure 5B).

p62/SQSTM1 binds autophagosomal membrane protein LC3, bringing SQSTM1-containing protein aggregates to the autophagosomes to be degraded [33]. The p62/SQSTM1 and neighbor of BRCA1 (NBR1) proteins promote autophagic degradation of ubiquitinated targets. This study examined the induction of p62/SQSTM1 and NBR1 in 7β-hydroxycholesterol-challenged macrophages. 7β-Hydroxycholesterol induced p62/SQSTM1 and NBR1 in a similar manner to LC3 induction (Figure 5A). Such induction was significantly potentiated by α-asarone (Figure 5B). Thus, α-asarone may enhance the specific interaction among LC3, p62/SQSTM1 and NBR1 for the formation and the degradation of polyubiquitin-containing bodies by autophagy.

**Figure 6:**
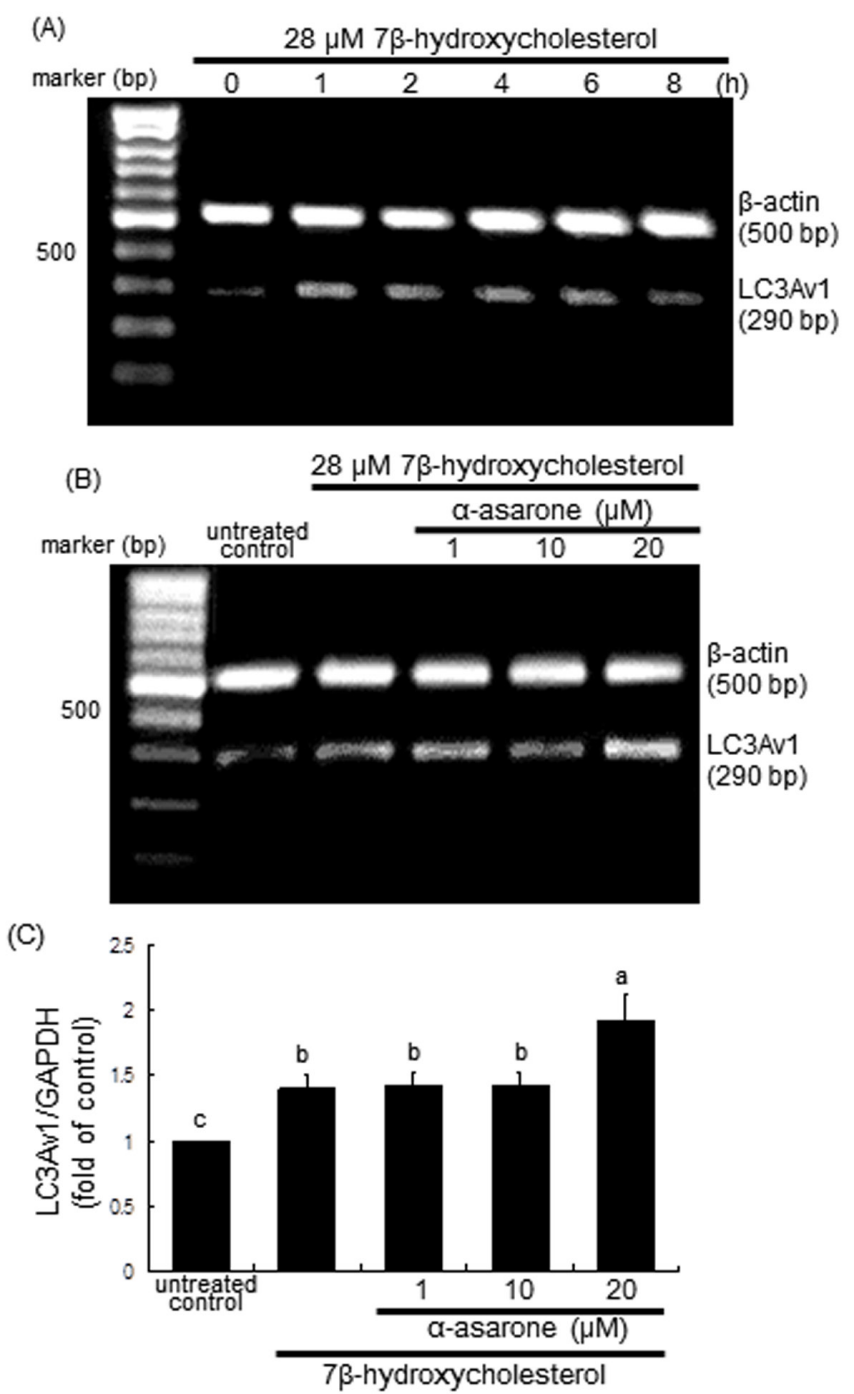
**RT-PCR A. and B., and real-time PCR C. data showing the time course response of LC3Av1 transcription to 7β-hydroxycholesterol A. and its elevation by α-asarone B. and C**. J774A1 cells were incubated with 28 μM 7β-hydroxycholesterol up to 8 h **A**., and in another set of experiments cells were incubated with 1-20 μM α-asarone and exposed to 28 μM 7β-hydroxycholesterol for 2 h **B**. and **C**. The LC3Av1 transcriptional levels were measured by RT-PCR and real-time PCR assays, and β-actin and GAPDH genes were used for the internal control. The bar graphs (mean ± SEM, *n* = 3) represent LC3Av1/GAPDH ratio. Values in bar graphs not sharing a letter indicate significant different at *P* < 0.05.

This study attempted to examine the association between autophagy and human LC3 protein during 7β-hydroxycholesterol- or α-asarone-induced autophagy in macrophages. RT-PCR analysis showed that the transcriptional variant of the LC3A gene LC3Av1, one of human LC3 gene family, was promptly activated at the transcriptional level in 7β-hydroxycholesterol-challenged macrophages (Figure 6A). However, its activation was further elevated due to treatment of macrophages with 20 μM α-asarone, confirmed by RT-PCR and real-time PCR analyses (Figure 6B and 6C).

### α-Asarone potentiation of Atg protein induction by 7β-hydroxycholesterol

**Figure 7:**
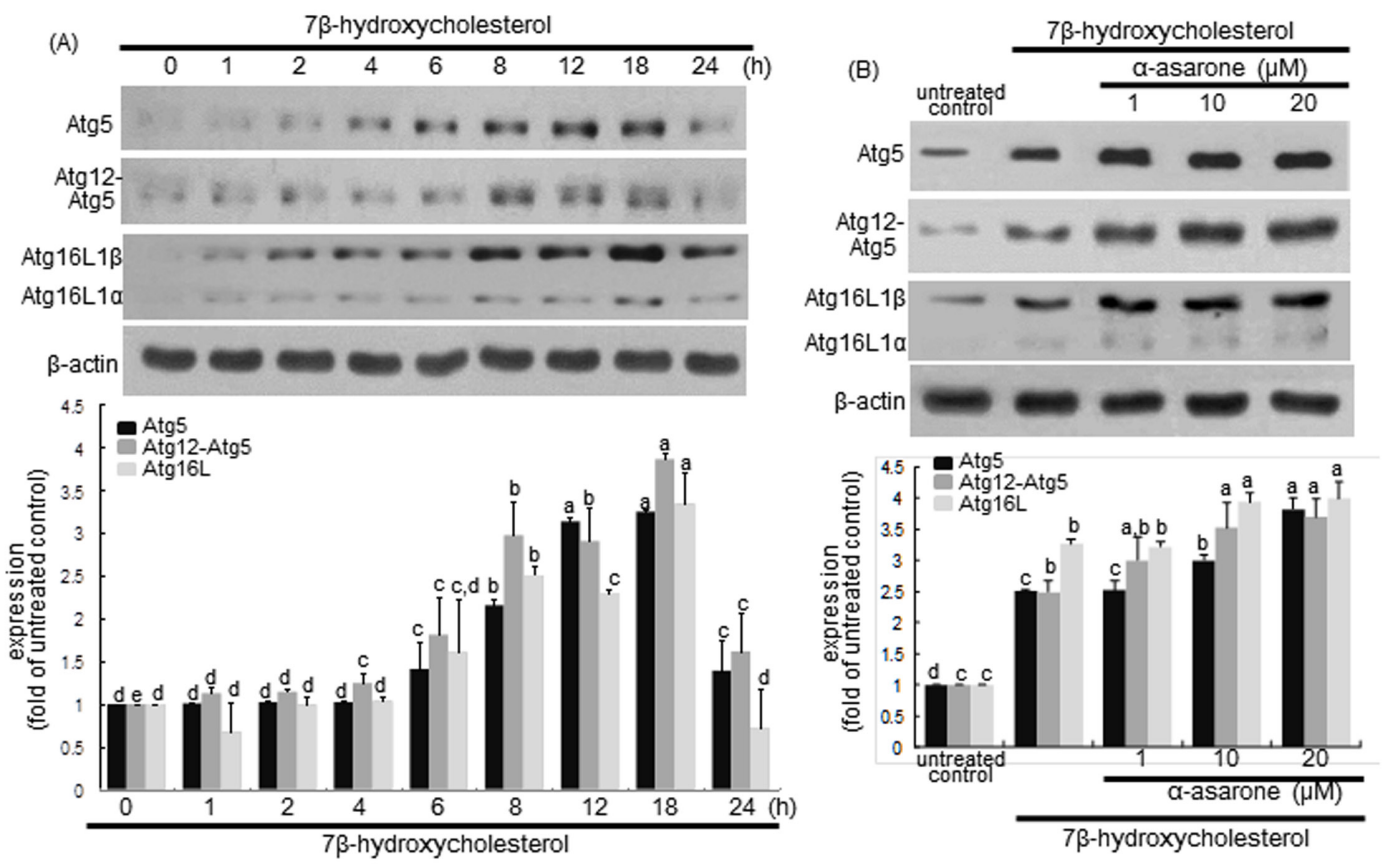
**Effects of 7β-hydroxycholesterol A. and α-asarone B. on induction of Atg5, Atg12-Atg5, and Atg16L1.** J774A1 macrophages were incubated with 28 μM 7β-hydroxycholesterol in the absence and presence of 1-20 μM α-asarone for various times. Cells were lysed and subject to electrophoresis on 12% SDS-PAGE and western blot analysis with a primary antibody of Atg5, Atg12-Atg5, or Atg16L1. β-Actin was used for the internal control. The bar graphs (mean ± SEM, *n* = 3) in the bottom panels represent quantitative results obtained from a densitometer. Values in bar graphs not sharing a letter indicate significant different at *P* < 0.05.

The process of autophagy can be divided into four steps such as induction, nucleation, vesicle expansion and closure, and autolysosome formation, which is regulated by the co-ordinated action of a number of Atg proteins [34]. The expansion of the developing autophagosomes is mediated by the Atg12-Atg5-Atg16L complex in an ubiquitin-like conjugation reaction [35]. Western blot data showed that 7β-hydroxycholesterol highly enhanced the protein levels of Atg5 and Atg16L in macrophages from 8 h up to 18 h after its treatment (Figure 7A). In addition, the level of Atg12-Atg5 conjugate was elevated in a similar fashion to Atg5. The levels of Atg5, Atg12-Atg5 conjugate and Atg16L were further enhanced in α-asarone-treated macrophages (Figure 7B). These results indicate that α-asarone augmented the expansion of phagophores by promoting the formation of Atg12-Atg5-Atg16L complex.

**Figure 8:**
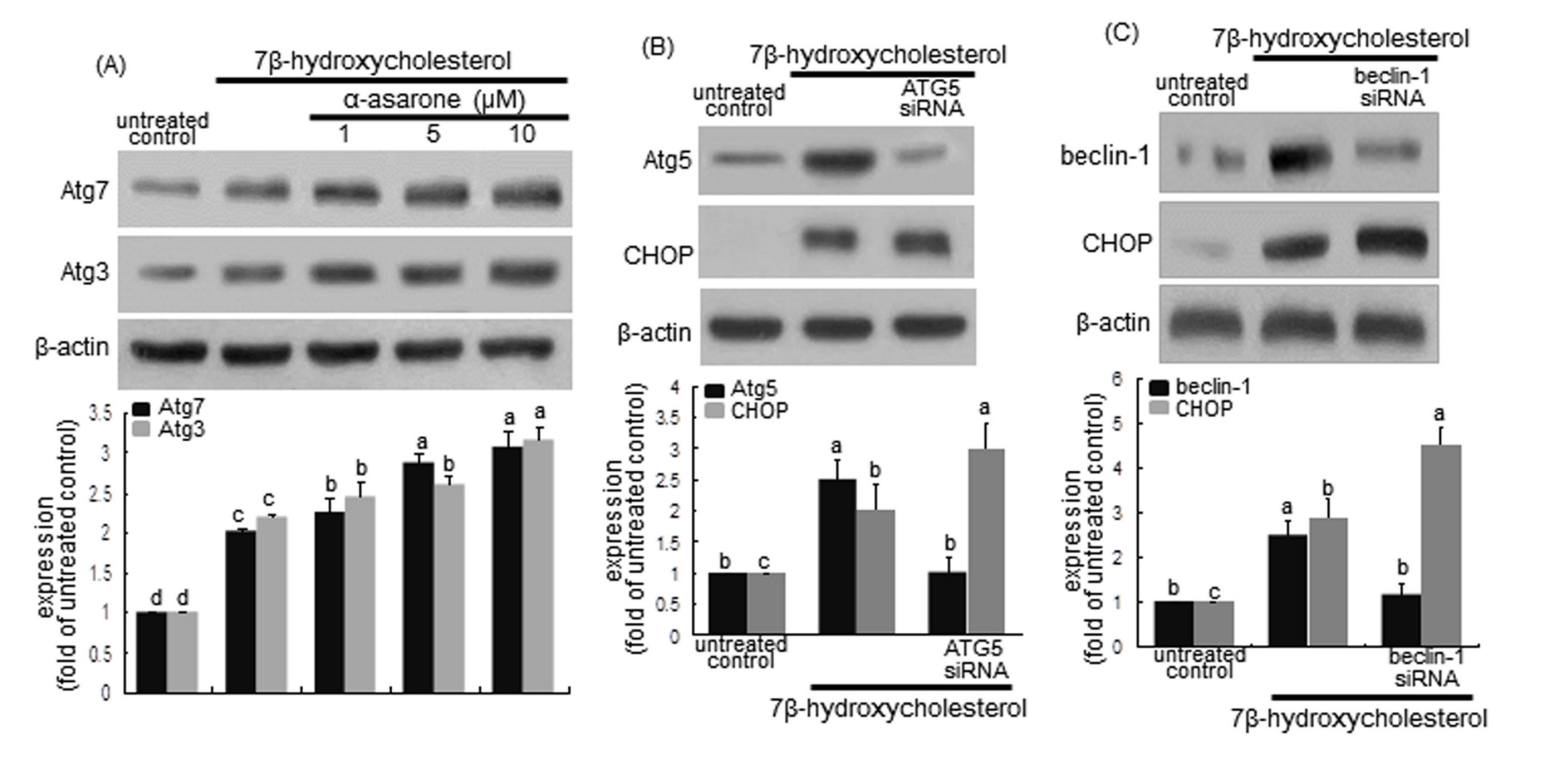
**Induction upregulaton of Atg3 and Atg7 by α-asarone A. and effect of Atg5 deletion B. and beclin-1 knockout C. on CHOP induction**. J774A1 macrophages were incubated with 28 μM 7β-hydroxycholesterol in the absence and presence of 1-20 μM α-asarone for various times. Cells were lysed and subject to electrophoresis on 12% SDS-PAGE and western blot analysis with a primary antibody of Atg3 or Atg7. β-Actin was used for the internal control. For the knockout of Atg5 gene or beclin-1 gene **B**., Atg5 siRNA or beclin-1 siRNA was introduced. The bar graphs (mean ± SEM, *n* = 3) in the bottom panels represent quantitative results obtained from a densitometer. Values in bar graphs not sharing a letter indicate significant different at *P* < 0.05.

LC3 is cleaved by Atg4 and bound to a lipid moiety of phosphatidylethanolamine by Atg3 and Atg7 to produce LC3II in another ubiquitin-like reaction [32, 35, 36]. When ≥1 μM α-asarone was treated to macrophages, the induction of Atg3 and Atg7 by 7β-hydroxycholesterol was potentiated in a dose-dependent manner (Figure 8A). Accordingly, α-asarone may facilitate the closure of the autophagosome through controlling LC3 lipidation.

The targeted deletion of Atg5 gene in macrophages using Atg5 siRNA increased the CHOP induction by 7β-hydroxycholesterol (Figure 8B). In addition, the knockout of beclin-1 gene in 7β-hydroxycholesterol-challenged macrophages induced the CHOP expression (Figure 8C). These results revealed that the inhibition of autophagy enhanced 7β-hydroxycholesterol-induced ER stress leading to macrophage apoptosis.

## DISCUSSION

**Figure 9:**
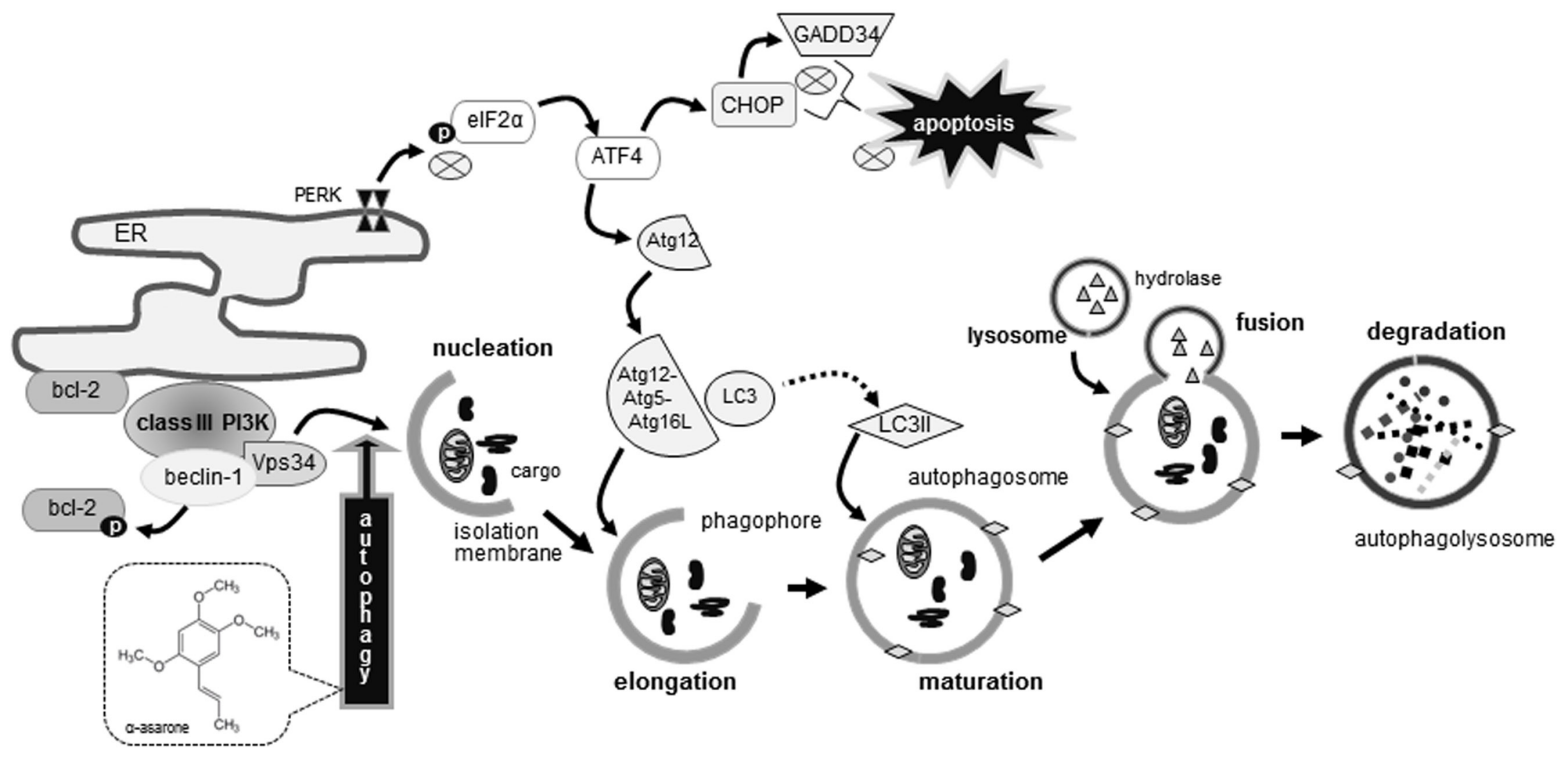
Schematic diagram showing the inhibitory effects of α-asarone on oxysterol-induced macrophage apoptosis As depicted, α-asarone blocked apoptotic elF2α process and enhanced beclin-1-dependent autophagy responsible for autophagolysosome formation. The symbol ⊗ indicates sites of inhibition manifested by α-asarone, while arrows designate activation.

Seven major findings were observed from this study. 1) The temporal response of eIF2α phosphorylation and GADD34 induction was dose-dependently down-regulated by treating macrophages with 1-20 μM α-asarone. 2) The α-asarone treatment increased autophagic vacuoles with distinct dot-like punctate structures localizing in the perinuclear regions of 7β-hydroxycholesterol-exposed macrophages. 3) The induction of beclin-1, Vps34 and p150, all responsible for vesicle nucleation, was further enhanced in ≥10 μM α-asarone-treated and 7β-hydroxycholesterol-exposed macrophages. 4) α-Asarone restored bcl-2 induction in 7β-hydroxycholesterol-exposed ER, whereas the bcl-2 phosphorylation in ER was enhanced by α-asarone, commencing autophagy process *via* vesicle nucleation by beclin-1-Vps34 complex. 5) The conversion of LC3I to LC3II and the induction of p62 and NBR1 were salient in α-asarone treated to macrophages exposed to 7β-hydroxycholesterol, enhancing autophagosome formation. 6) The level of Atg12-Atg5 conjugate and the induction of Atg16L were elevated in α-asarone-treated macrophages, indicating that this compound promoted the expansion of phagophores to form autophagosomes. 7) The induction of Atg3 and Atg7 by 7β-hydroxycholesterol was potentiated by α-asarone, facilitating the closure of the autophagosome through controlling LC3 lipidation. Therefore, α-asarone enhanced ER stress-mediated macrophage autophagy through inhibiting eIF2α-CHOP pathway-mediated macrophage apoptosis induced by 7β-hydroxycholesterol (Figure 9).

The UPR is mediated by cellular signals through three transmembrane sensors of protein kinase RNA-like ER kinase (PERK), inositol requiring kinase/endonuclease-1α (IRE-1α) and activating transcription factor 6 (ATF6). These three canonical response pathways results in the inhibition of misfolded protein translation, and facilitates protein degradation of ER components for normal ER folding setting [37]. There is now ample evidence that ER stress and UPR are chronically activated in atherosclerotic macrophages and endothelial cells [38, 39]. In particular, pro-atherogenic effect of prolonged ER stress is the activation of inflammatory pathways in macrophages [37]. Macrophages are vulnerable to lipid-induced toxicity in the setting of metabolic diseases, which leads to drive macrophages toward apoptosis [37]. One investigation shows that CD36-mediated oxidized LDL uptake triggers ER stress response in macrophages, enhancing the foam cell formation [40]. In our previous study, 7β-hydroxycholesterol resulted in ER stress-mediated macrophage apoptosis through pathways involving activation of the ER IRE1α and PERK [28]. In addition, α-asarone prevented ER stress induced-apoptosis by interfering the IRE1α downstream signaling and by disturbing PERK-ATF4 pathway in 7β-hydroxycholesterol-experienced J774A1 macrophages.

The PERK phosphorylates eIF2α and regulates ATF4 transcriptional activity to attenuate protein translation as a defensive mechanism of UPR [41]. Accordingly, the PERK-phospho-eIF2α-ATF4 signaling inhibits the decline of protein synthesis during chronic ER stress by stimulating signaling downstream of the mammalian target of rapamycin complex 1 [42]. One study shows that the eIF2 phosphorylation is involved in polyglutamine 72 repeat aggregates-induced LC3 conversion [43]. The malfolded proteins induced ER stress-mediated cell death through PERK-eIF2α phosphorylation, which was inhibited by autophagy formation involving LC3 conversion and aggregate degradation. This study also showed that 7β-hydroxycholesterol induced both eIF2α phosphorylation and LC3 conversion in macrophages, indicating that this oxysterol generated malfolded proteins leading to ER stress. One can assume that when LC3 conversion and autophagy formation are not enough to diminish malfolded proteins, cells may undergo ER stress-mediated cell death with caspase-12 activation [43]. Congruently, 7β-hydroxycholesterol induced ER stress-mediated macrophage apoptosis with caspase-12 activation [28]. Additionally, α-asarone abrogated ER stress induced-cell death by decreasing the cleavage of caspase-12. Accordingly, α-asarone sufficiently enhanced the LC3 conversion for the autophagy formation enough to reduce formation of malfolded proteins triggered by 7β-hydroxycholesterol.

The autophagic alteration has been considered as a potential therapeutic target for diverse diseases, including neurodegenerative diseases, cancers and infectious diseases [6, 9, 44]. The implication of autophagy in human diseases has developed small-molecule modulators and pharmacologic agents with distinctive molecular targets in different human pathologies [8]. A variety of therapeutic agents target specific molecular components of the core autophagic machinery. Inhibiting autophagy is a promising approach in cancer therapy, based on evidence that autophagy is a survival-promoting mechanism in cancer cells, whereas the induction of autophagy is associated with the resistance of cancer cells to chemotherapeutic agents [45]. Accumulating evidence demonstrates that the induction of autophagy is a neuroprotective response in the context of neurodegenerative disorders such as Alzheimer disease, Huntington's disease and Parkinson's disease [44, 46]. In addition, it is deemed that the activation of autophagy is cardioprotective, whereas excessive autophagy can lead to cell death and cardiac atrophy [47]. Accordingly, the alterations of the key proteins in the core autophagy machinery and upstream regulators represent an attractive therapeutic target for treating diverse diseases.

Recent studies imply that autophagy can be induced by dietary polyphenols such as resveratrol, catechin, oleuropein and curcumin [22, 23, 48-50]. EGCG reduces intracellular lipid accumulation by stimulating LC3 conversion-associated autophagy through a Ca^2+^/calmodulin-dependent protein kinase kinase β-mediated mechanism [24]. Curcumin induces a beneficial form of autophagy in H_2_O_2_-exposed human vascular endothelial cells *via* a protective mechanism involving FOXO1, which may be a potential therapeutic avenue for the treatment of oxidative stress-related cardiovascular diseases [51]. Resveratrol induces autophagy in human dermal fibroblasts through regulating death-associated protein kinase 1, confirming the beneficial effects of resveratrol on autophagy in skin [52]. This stilbene suppresses autophagy-induced apoptosis in human U251 glioma cells [49]. Similarly, in the current study α-asarone increased autophagy induced by 7β-hydroxycholesterol in macrophages through negatively influencing eIF2-GADD34-CHOP-dependent mechanism, suggesting the therapeutic effects of α-asarone on the inhibition of macrophage cell death. In addition, α-asarone enhanced the bcl-2 phosphorylation in ER of oxysterol-treated macrophages, which appeared to hamper the binding of beclin-1 stimulating autophagic activity. In our previous study, α-asarone dampened 7β-hydroxycholesterol-induced macrophage apoptosis through blocking ER stress-specific signaling involving caspase-12 activation [27]. Taken together, α-asarone may be an anti-atherosclerotic multi-targeted agent antagonizing eIF2α-GADD34-CHOP-mediated macrophage apoptosis and concurrently inducing pro-survival autophagy involving beclin-1 signaling pathway.

## CONCLUSIONS

The current report demonstrated that α-asarone abrogated 7β-hydroxycholesterol-triggered eIF2α-CHOP activation, and enhanced macrophage autophagy through up-regulating autophagolysosome formation. α-Asarone boosted the beclin-1-Vps34-p150 induction for the phagophore elongation and the LC3 conversion for the membrane lipidation promoted by 7β-hydroxycholesterol, both required for the expansion of phagophores to form autophagosomes. Although α-asarone may serve as an effective in stimulating macrophage autophagy to degrade malfolded proteins in ER possibly generated by oxysterols, animal and clinical studies are required to investigate the *in vivo* effectiveness of α-asarone.

## MATERIALS AND METHODS

### Materials

Dulbecco's Modified Eagle Medium (DMEM) chemicals, monodansylcadaverine (MDC) and 7β-hydroxycholesterol were obtained from Sigma Aldrich Chemical (St. Louis, MO) as were all other reagents, unless specifically stated elsewhere. Fetal bovine serum (FBS) and penicillin-streptomycin were provided by Lonza (Basel, Switzerland). α-Asarone was purchased from Cayman chemical company (Ann Arbor, MI). Antibodies of eIF2α, phospho-eIF2α, Vps34, Atg5, Atg12-Atg5, Atg16L1, SQSTM1, NBR1, Atg7, Atg3, CHOP were obtained from Cell Signaling Technology (Danvers, MA). GADD34, beclin-1 antibodies were purchased from Abcam (Cambridge, UK). p150 antibody was obtained from Santa Cruz Biotechnology (Dallas, TX). LC3 antibody was purchased from MBL international corporation (Woburn, MA). Horseradish peroxidase (HRP)-conjugated goat anti-rabbit and rabbit anti-mouse IgG were supplied from Jackson ImmunoResearch Laboratory (West Grove, PA).

### Cell culture

Mouse macrophage cell line J774A1 was obtained from American Type Culture Collection (ATCC; Manassas, VA) and grown in DMEM supplemented with 10% FBS at 37°C in a humidified atmosphere of 5% CO_2_ in air. Murine macrophages were treated with 1-20 μM α-asarone and exposed to 28 μM 7β-hydroxycholesterol for various times. In culture experiments, J774A1 macrophages were incubated in DMEM supplemented with 0.4% fatty acid-free bovine serum albumin (BSA).

The cytotoxicity of α-asarone was determined by using MTT (3-(4,5-dimethylthiazol-2-yl)-2,5-diphenyltertrazolium bromide) assay. Cells treated with α-asarone for 24 h were incubated with 1 mg/ml MTT solution at 37°C for 3 h, resulting in the formation of insoluble purple formazan product that was dissolved in 250 µl isopropanol. Optical density was measured using a microplate reader at wavelength 570 nm. This study found that α-asarone at the doses of 1-20 μM had no cytotoxicity (Figure 1B). The current experiments employed α-asarone in the range of 1-20 μM.

### ER isolation

After macrophages were treated with 28 μM 7β-hydroxycholesterol and 20 μM α-asarone, the ER isolation was conducted with a commercial ER Enrichment kit (Novus Biological, Littleton, CO), according to the procedure suggested by the manufacture. Briefly, cells were lysed with hypotonic extraction buffer [10 mM HEPES (pH 7.8), 25 mM KCl, 1 mM EGTA] and centrifuged at 600 g for 5 min. Pellets were lysed with isotonic extraction buffer [10 mM HEPES (pH 7.8), 250 mM sucrose, 25 mM KCl, 1 mM EGTA], followed by homogenization. After centrifugation at 10,000 g for 30 min, the supernatants were rough ER fraction. The ER fractions were subject to western blot analysis for the detection of specific proteins.

### Western blot analysis

Following culture protocols, J774A1 cells were extracted in a lysis buffer containing with 10 mg/ml β-glycerophosphate, 0.1 M Na_3_VO_4_, 0.5 M NaF and protease inhibitor cocktail. Equal amounts of protein were electrophoresed on 8-15% SDS-PAGE and transferred onto a nitrocellulose membrane. After blocking with 5% non-fat skim milk or 3% BSA for 3 h at room temperature, the membranes were incubated with polyclonal or monoclonal antibodies of eIF2α, phospho-eIF2α, GADD34, beclin-1, Vps34, p150, Atg5, Atg12-Atg5, Atg16L1, p62/SQSTM1, NBR1, LC3, Atg7, Atg3, and CHOP for overnight at 4°C. After three times of washing with Tris buffered saline-tween 20 buffer, the membranes were incubated with anti-rabbit or anti-mouse IgG conjugated to HRP for 1 h at room temperature. The individual protein level was detected by Immobilon Western Chemiluminescent HRP substrate (Millipore, Billerica, MA). For the internal control, the membranes were incubated with β-actin antibody (Sigma Aldrich Chemicals). After the performing immunoblot analysis, the blot bands were visualized on Agfa X-ray film (Agfa-Gevaert, Belgium), developing signals with X-ray developer and fixer (Duksan, Seoul, Korea).

### Autophagolysosome staining

After J774A1 macrophages were treated with 20 μM α-asarone and exposed to 28 μM 7β-hydroxycholesterol for 18 h, cells were fixed with 4% formaldehyde for 20 min. After the fixation of J774A1 macrophages, cells were incubated with 50 μM MDC (melt in methanol) and 50 μM acidotropic probe LysoTracker^®^ Red DND-99 (Life Technologies, Waltham, MA) for 1 h at 37°C and washed with phosphate buffered saline (PBS)-tween 20. For the nuclear staining, cells were incubated with 4’,6-diamidino-2-phenylindole (DAPI) for 10 min at 37°C. After the several washes with PBS-tween 20, cells mounted on a slide glass were visualized by fluorescence confocal microscopy (Carl Zeiss, Oberkochen, Germany).

### RT-PCR and real-time PCR analyses

Total RNA was extracted from J774A1 macrophage lysed for 5 min using a commercial Trizol reagent kit (Molecular Probes Inc., Cincinnati, OH). The complementary DNA was synthesized using 5 μg of total RNA with 200 units of reverse transcriptase (Promega Corp. Madison, WI) and 0.5 mg/ml oligo-(dT)_15_ primer (Bioneer, Daejeon, Korea).

### RT-PCR

The PCR was conducted using mRNA transcripts of mouse LC3A variant 1 (LC3Av1, forward primer: 5’-GACCAGCACCCCAGTAAGAT-3’ and reverse primer: 5’-AAGCCGAAGGTTTCTTGGGA-3’, product size: 290 bp) and β-actin (forward primer: 5’-GACTACCTCATGAAGATC-3’, reverse primer: 5’-GATCCACATCTGCTGGAA-3’, product size: 512 bp) with addition of 25 μl of 10 mM Tris-HCl buffer (pH 9.0) containing 25 mM MgCl_2_, 10 mM dNTP and 5 units of Taq DNA polymerase (Takara Bio Inc., Minamikusatsu, Japan). The PCR conditions of LC3Av1 and β-actin were established sequentially as pre-denaturation at 94°C for 3 min, and 30 cycles of 94°C for 30 s, annealing at 59°C for 45 s and elongation at 72°C for 90 s, and the final extension was done at 72°C for 10 min. After thermocycling and electrophoresis of the final PCR products on 1% agarose gel containing 0.1% ethidium bromide. Each band was visualized by UV transilluminator (Vilber-Lourmat, Marne-la-Vallee, France) and gel photographs were obtained.

### Real-time PCR

The level of mRNA transcripts of LC3Av1 was also quantified by real-time PCR using a SYBR Green PCR commercial kit (Qiagen, Valencia, CA). Primers for LC3Av1 (forward primer: 5’- TGGGAGGCGTAGACCATGTA-3’, reverse primer: 5’-TTGGTCAAGATCCGGCG-3’, product size: 145 bp) were used. The housekeeping gene GAPDH (forward primer: 5’- CCTCCTGTTCGACAGTCAGC-3’, reverse primer: 5’-CGCCCAATACGACCAAATCC-3’, product size: 113 bp) was used for an internal normalization for the coamplification. Quantitative PCR was carried out at 95°C for 3 min, followed by 40 cycles of denaturation at 95°C for 10 s, annealing at 62°C for 15 s and extension at 72°C for 30 s.

### Atg5 small interfering RNA (siRNA) transfection

For the deletion of Atg5 gene, a major biomarker of autophagy, Atg5 siRNA transfection assay was conducted with J774A1 cells using a commercial lipofectamine 3000 mixture (Life Technologies). Cells were incubated with 5 μg Atg5 siRNA (Thermo Scientific, Waltham, MA) and lipofectamine 3000 mixture for 4 h at 37°C. After the transfection, J774A1 cells were treated with 28 μM 7β-hydroxycholesterol for 18 h. Subsequently, cells were extracted in a lysis buffer and western blot analysis was performed with Atg5 antibody for the detection of the Atg5 deletion.

### Statistical analysis

The results are presented as means ± SEM. Statistical analysis was conducted using the SAS software package version 6.12 (SAS Institute, Cary, NC). One-way ANOVA was used to determine boosting effect of α-asarone on 7β-hydroxycholesterol induced-autophagy in macrophages. Differences among treatment groups were analyzed with Duncan's multiple range test and considered significant at P < 0.05.

## Abbreviations

Atg: autophagy related protein
CHOP: C/EBP homologous protein
eIF2α: eukaryotic initiation factor 2α
ER: endoplasmic reticulum
GADD34: growth arrest and DNA damage-inducible protein 34
LC3: microtubule-associated protein 1 light chain 3
LC3Av1: LC3A variant 1
NBR1: neighbor of BRCA1
PE: phosphatidylethanolamine
PI3K: phosphatidyinositol-3 kinase
SQSTM1: sequestosome 1
UPR: unfolded protein response
Vps34: vacuolar protein sorting 34
